# Posterior Mitral Valve Annulus Vegetation in the Presence of Mitral Annulus Calcification: A Case Report

**DOI:** 10.7759/cureus.32283

**Published:** 2022-12-07

**Authors:** Andreas Kyvetos, Panagiota Voukelatou, Ioannis Vrettos, Tasos Nikas, Anastasia Manoli, Elektra Papadopoulou

**Affiliations:** 1 2nd Department of Internal Medicine, General and Oncology Hospital of Kifissia “Agioi Anargyroi”, Athens, GRC; 2 2nd Pediatric Department, Pendelis General Children’s Hospital, Athens, GRC; 3 1st Cardiology Department, Evangelismos General Hospital, Athens, GRC

**Keywords:** modified duke’s criteria, bioprosthetic, pet-ct with 18f-fdg, mitral annulus calcification, endocarditis

## Abstract

The evaluation of patients who present at the emergency department with fever and a history of cardiac valve replacement should be thorough, and the possibility of endocarditis must be high in the differential diagnosis. The modified Duke’s criteria are recommended for the diagnosis of endocarditis, and the role of positron emission tomography-computed tomography (PET-CT) scan is highlighted in the presence of bioprosthetic valves among the recent guidelines. Here, we describe a challenging case of endocarditis in a patient with severe mitral annulus calcification and bioprosthetic aortic valve replacement. Transesophageal echocardiography revealed an echogenic mass on the posterior mitral annulus, which was confirmed to be a vegetation on the PET-CT scan. Despite adequate antibiotic therapy and no indication for emergency cardiac surgery, in the fourth week of treatment, an embolic event in the ophthalmic artery occurred, and the patient was admitted for surgery. Intraoperatively, the presence of vegetation was confirmed. Because severe mitral annulus calcification may act as a nidus for infective endocarditis, special attention must be paid to these patients. Additional studies are required in patients with residual vegetation at the end of antibiotic treatment, especially if they have increased dimensions, to accurately formulate the optimal management plan.

## Introduction

Infective endocarditis (IE) is the infection of a heart valve or endocardial surface; however, sometimes, the site of the vegetation, the hallmark of IE, is atypically located. The diagnosis is based on the modified Duke’s criteria. Nevertheless, the diagnostic workup remains challenging, and special attention is needed as IE has a high mortality rate. The cornerstone for the diagnosis of endocarditis is the echocardiogram (ECG). However, a negative result is common, especially in the early stages of the disease, with the current guidelines suggesting repeating it in 7-10 days. Although vegetations have typical sites on ECG that are useful for differential diagnosis, vegetation can be found anywhere on the components of the valvular and subvalvular apparatus. Newer diagnostic tools, such as positron emission tomography-computed tomography (PET-CT) scans, are very useful for the diagnosis and follow-up of these patients [1].

## Case presentation

We present the case of a febrile 78-year-old Caucasian female patient with a history of bioprosthetic aortic valve replacement seven years ago (Magna ease 21), type 2 diabetes mellitus, and chronic kidney disease. Her home medications included metformin (850 mg twice a day). She presented at the emergency department with a 20-day history of intermittent fever, without seeking any medical assistance until that time. She reported that her fever was above 38.5°C, mainly in the morning and at night, and subsided with the use of paracetamol. She experienced seven days of consistent fever followed by seven days of normothermia. The week before the presentation she had a fever with daily recurrent paroxysms.

**Figure 1:**
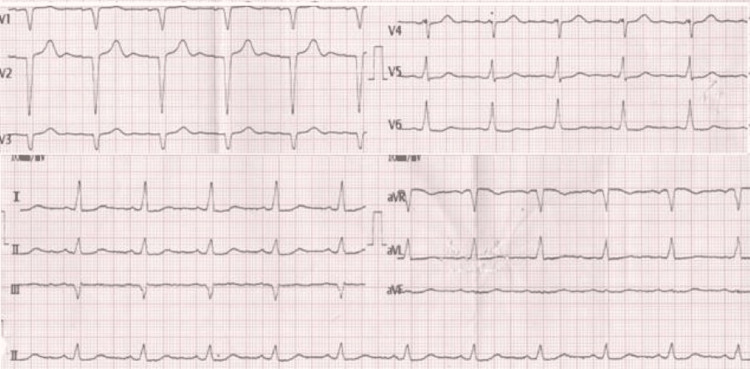
Echocardiogram on admission showing normal sinus rhythm with no ischemic lesions.

She denied any other symptoms including abdominal pain, diarrhea, dysuria, headaches, arthralgias, myalgias, fatigue, weight loss, or visual disturbances. She had no history of alcohol or illicit drug use. She did not report any allergies. There was no family, sexual, or travel history of note, and she had no contact with animals. On examination, her consciousness was clear and she did not have any breathing difficulty at rest, with a respiratory rate of 18 breaths per minute. Her pulse oximetry was 99%, her blood pressure was 145/103 mmHg, and her body temperature was 38°C. Cardiac auscultation revealed a 4/6 holosystolic murmur through the precordium. She had no signs indicating urinary or abdominal infection. The neurological examination was unremarkable, her temporal arteries were palpable, and skin rashes were absent. The ECG on admission showed sinus rhythm, 1:1 conduction of about 90 beats per minute, and no signs of ischemic lesions (Figure 1).

**Figure 2:**
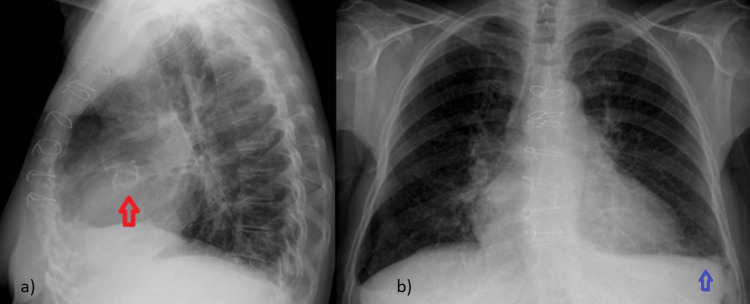
Plain chest radiograph images. (a) Lateral and (b) posteroanterior images reveal blunting of the left costophrenic angle (blue arrow) and the silhouette of the aortic valve replacement (red arrow). Additionally, cardiomegaly is observed.

The chest plain radiograph revealed blunting of the left costophrenic angle and cardiomegaly (Figure 2).

Initial laboratory analyses showed an abnormal erythrocyte sedimentation rate and elevated C-reactive protein (CRP) level (Table 1).

**Table 1:** Initial laboratory analyses. WBC = white blood cell count; HCT = hematocrit; Hb = hemoglobin, ESR = erythrocyte sedimentation rate; Cr = creatinine; CRP = C-reactive protein; hs-TNI = high-sensitivity troponin I

Parameter	Value	Normal range
WBC	10.58 × 10^3^/μL	4.0–11.0 × 10^3^/μL
HCT	31.4%	37–48%
Hb	10.2 g/dL	12–16 g/dL
ESR	111 mm/hour	2–20 mm/hour
Cr	2.2 mg/dL	0.6–1.4 mg/dL
CRP	11.2 mg/dL	<0.5 mg/dL
D-dimer	3,206.7 μg/dL	<500 μg/dL
hs-TNI	29 pg/mL	<11.6 pg/mL

At this time, infections, malignancies, autoimmune, and inflammatory diseases were considered among the possible diagnoses. Three blood cultures were obtained on the first day, spacing the venipunctures at least 30 minutes apart. The results for hepatitis B virus, hepatitis C virus, Epstein-Barr virus, cytomegalovirus, and *Toxoplasma* infection were unremarkable. Rheumatoid factor, procalcitonin, C3, C4, and quantitative determination of immunoglobulins were within normal limits. Urine culture was negative.

**Figure 3:**
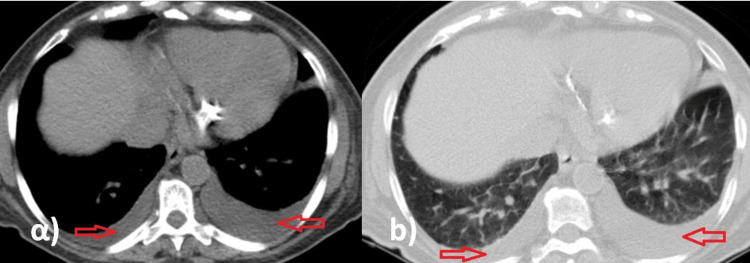
Chest computed tomography scan images. (a) Mediastinal window and (b) lung window reveal bilateral pleural effusion (red arrows).

Full body computed tomography (CT) scans were scheduled and a further diagnostic workup was planned, including a diagnostic panel for autoimmune and infectious diseases, serum angiotensin-converting enzyme, immunoelectrophoresis, and quantiferon test, as these examinations were not available in our hospital. The next day, three out of three blood cultures were positive for *Streptococcus gallolyticus* subspecies *pasteurianus*. All other tests, except CT, were postponed, and the diagnostic workup was focused to prove the suspected endocarditis. Cardiac transthoracic ultrasound was performed which revealed an ejection fraction of 55% with mild mitral valve stenosis and regurgitation with severe mitral annulus calcification and no signs of vegetation. Normal aortic bioprosthesis function was observed. A full-body CT scan was performed which revealed only a bilateral pleural effusion (Figure 3).

Transesophageal echocardiography (TEE) was performed, and no signs of endocarditis were noted. Fundoscopic examination revealed Roth spots. At this time, the patient had a definite diagnosis of endocarditis (on major and three minor criteria according to the modified Duke’s criteria [1]); hence, intravenous antibiotic therapy with ceftriaxone 2 g per day was initiated. Blood cultures were negative after three days of treatment, and the CRP level was back to normal.

**Figure 4:**
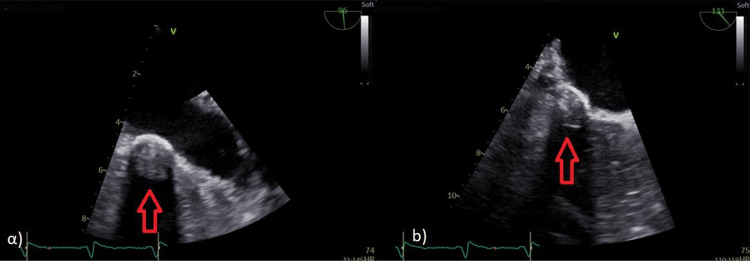
Transesophageal echocardiography. Red arrows show the echogenic mass on the posterior mitral annulus.

**Figure 5:**
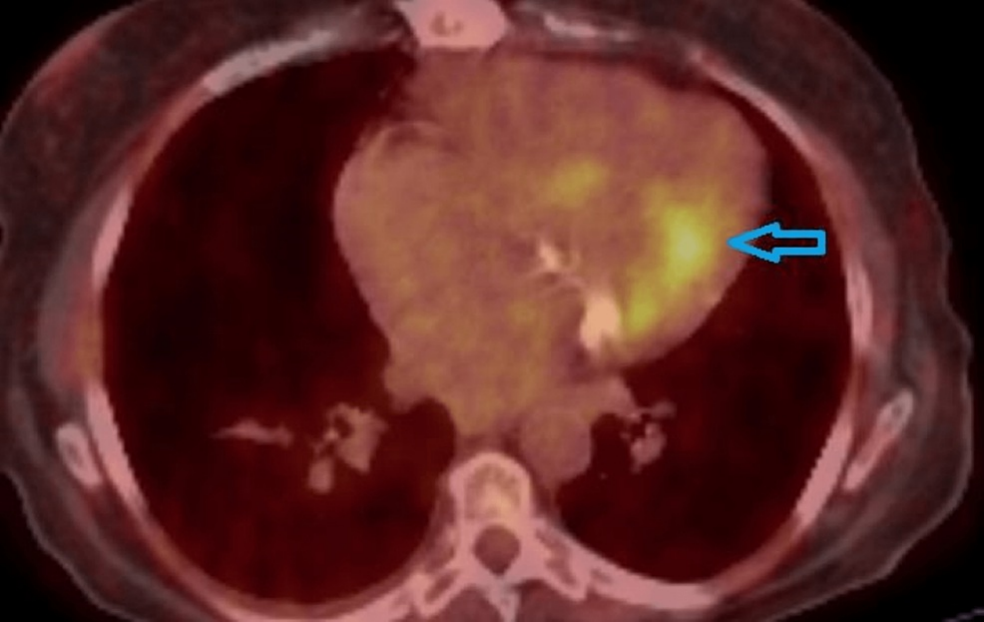
Positron emission tomography-computed tomography scan. The blue arrow shows the 18F-fluorodeoxyglucose uptake on the posterior mitral annulus (standardized uptake value max = 5.4).

Because of the history of bioprosthetic aortic valve replacement, a second TEE was performed on the third week of therapy which revealed an echogenic mass of 12 × 13 mm attached to the posterior mitral annulus (Figure 4). At this time, the diagnosis of a vegetation was suspected in accordance with the patient’s history. A PET-CT scan was performed because of the unusual site of the vegetation that confirmed our hypothesis (Figure 5).

Despite the well-controlled infection, after completing four weeks of antibiotic treatment, she had an embolic event in the ophthalmic artery. At this time, surgery for mitral valve replacement was decided. Intraoperatively, the presence of the vegetation was confirmed. The patient completed all follow-up visits for six months.

## Discussion

The diagnosis of IE is based on the modified Duke’s criteria in which the role of PET-CT scan is highlighted [1]. Despite the development of new diagnostic tools, the one-year survival rate of IE has not improved and is only 30% [2]. In our case, because one major and three minor criteria from Duke’s criteria were met, the diagnosis of IE was definite [1]. However, we did not know where the vegetation was placed, and, therefore, the duration of the treatment.

According to the European Society of Cardiology, echocardiography is recommended as the first-line imaging modality in suspected IE, but a negative result is observed in 15% of the cases. This is explained by the fact that vegetation may be very small, atypically located, or at the early stage of the disease [3]. The sensitivity of TEE is 96% for native and 92% for prosthetic valve endocarditis [1]; therefore, with a high suspicion for IE, it is recommended to repeat TEE within five to seven days.

Despite the first negative TEE, it was subsequently repeated in a tertiary center, and a hypogenic mass was detected attached to the posterior mitral annulus which was heavily calcified. In our case, despite the presence of a prosthetic aortic valve, the vegetation was found on the posterior mitral annulus, an atypical location. The PET-CT scan was performed and the diagnosis of IE of the posterior mitral annulus was confirmed.

Mitral annulus calcification is a chronic process that involves the annulus of the mitral valve [4]. The association between mitral annulus calcification and endocarditis is documented in some studies [5-7]. A case series showed that mitral annulus calcification is a possible nidus for endocarditis, especially for *Staphylococcus aureus* [8]. According to current guidelines, the duration of treatment for native valve endocarditis, caused by *Streptococcus gallolyticus* subspecies *pasteurianus*, is four weeks of monotherapy with ceftriaxone 2 g once daily [1]. Despite appropriate antimicrobial treatment, neurological complications are found in 20-40% of patients [9], and the risk of these events is higher in the mitral than in the aortic valve [10]. In our case, despite the well-controlled infection, the patient had an embolic episode of the ophthalmic artery after the completion of four weeks of therapy. Residual vegetation at the end of antibiotic treatment for IE is common. This is based on the fact that even if bacteria are eradicated, fibrin or platelet deposition may continue [11]. Hence, the natural progression of the vegetation under antibiotics remains unknown [11]. The prognostic value of residual vegetation is not clear. Caution is needed in large residual vegetations >10 mm, or in case they grow under treatment [11]. In our patient, despite the optimal antibiotic treatment according to guidelines, an embolic episode occurred that led her to the surgery room for mitral valve replacement.

## Conclusions

IE remains a significant diagnostic challenge, and PET-CT scan, as demonstrated in recent guidelines, plays a significant role in the diagnostic workup. Additional diagnostic tools, such as the PET-CT scan in collaboration with TEE, can identify with accuracy the site of the vegetation. Because mitral annulus calcification is a possible site for vegetation, it must be thoroughly inspected when endocarditis is suspected. Additional studies are required in patients with residual vegetation at the end of antibiotic treatment, especially if they have increased dimensions, to accurately formulate the optimal management plan.
